# A Single Clinical Case of Leptospirosis in a 70-Year-Old Man During the Military Conflict in Ukraine

**DOI:** 10.1089/vbz.2023.0007

**Published:** 2023-07-11

**Authors:** Olena Zubach, Iryna Pestushko, Yulia Dliaboha, Oksana Semenyshyn, Alexander Zinchuk

**Affiliations:** ^1^ Department of Infectious Diseases, Danylo Halytsky Lviv National Medical University, Lviv, Ukraine.; ^2^ Lviv Oblast Clinical Hospital of Infectious Diseases, Lviv, Ukraine.; ^3^ St. Panteleimon Hospital of the First Territorial Medical Association, Lviv, Ukraine.; ^4^ State Institution Lviv Oblast Center for Disease Control and Prevention of the Ministry of Health of Ukraine, Lviv, Ukraine.

**Keywords:** leptospirosis, leptospira, One Health, clinical case, Ukraine, military conflict

## Abstract

Leptospirosis is a bacterial disease that affects both humans and animals worldwide. Clinical symptoms of leptospirosis in humans range widely, from mild to severe illness, with symptoms that can include severe jaundice, acute renal failure, hemorrhagic pneumonia, and meningitis. We present a detailed clinical description of a 70-year-old man with leptospirosis. This case presented without the typical prodromal period for leptospirosis, thus making diagnosis more difficult. This isolated case occurred in the Lviv region during the ongoing military conflict between Russia and Ukraine where Ukrainian citizens have been forced to hide in premises that are not properly adapted for their long-term stay, which result in conditions that can potentially lead to the emergence of many infectious diseases. This case highlights the need for heightened awareness into the symptoms of a variety of infectious diseases, including but not limited to leptospirosis.

## Introduction

Leptospirosis in humans is an acute infectious disease characterized by fever, myalgia, damage to the kidneys, liver, nervous and cardiovascular system, and thrombohemorrhagic syndrome. The disease occurs resulting from direct or indirect exposure to infected reservoir host animals. Murine rodents are the main reservoir and source of infection, as they contain *Leptospira* in their renal system (Haake and Levett, 2015). Most often, human infections are a result of contact with contaminated water from rivers or stagnant lake water that contain rodent excrement (Levett, 2020; Picardeau, 2013).

Traditionally, the leptospirosis incidence is highest in regions or countries with a tropical or subtropical climate, such as Oceania (150.68 cases per 100,000 population), Sri Lanka (52.12 cases per 100,000), and Malaysia (30.2 cases per 100,000) (Fann et al., 2020; Guernier et al., 2011; Warnasekara et al., 2019). These regions are characterized by year-round high temperatures accompanied with significant humidity from heavy rainfall during the rainy season, resulting in frequent floods (Hacker et al., 2020). The occurrence of floods worsens sanitary and hygienic conditions and promotes rodent reproductive potential, which inevitably causes an increase in leptospirosis cases among humans (Chadsuthi et al., 2021).

In countries with temperate continental climates such as Ukraine, the incidence of leptospirosis is significantly lower, with cases predominantly associated with specific professional activities (Pryshliak et al., 2020; Ukhovskyi et al., 2018). These professions include but are not limited to plumbers, fishermen, miners, and professional athletes (Goarant, 2016; PHE, 2018; Walker, 2018). These professions are often in contact with standing water and thus have a higher risk for leptospirosis exposure. In these cases, the transmission of leptospirosis most often occurs through mucous membranes and skin wounds/sores (WHO, 2022a).

Despite a lower prevalence of leptospirosis cases in Ukraine compared with regions with a subtropical climate, leptospirosis cases are registered annually. In 2019, 295 cases of leptospirosis were officially registered in Ukraine (0.7 per 100,000); in 2020, 120 cases (0.28 per 100,000); in 2021, 122 cases (0.29 per 100,000); and in 2022, 141 cases (0.34 per 100,000) (Public Health Centre of Ukraine, 2022). The real incidence of leptospirosis in Ukraine is likely higher than reported values due to the underdiagnoses and under-reporting of human leptospirosis cases (Zubach et al., 2020).

The clinical symptoms of leptospirosis can vary widely, making disease diagnosis very challenging. Leptospirosis is typically characterized by a pronounced prodromal period in which patients experience increasing fever, headache, vomiting, diarrhea, and myalgia (especially in calf muscle). As the disease progresses, patients develop multiple organ dysfunction, including acute renal failure, jaundice, lung involvement with the development of acute respiratory distress syndrome, aseptic meningitis, and thrombohemorrhagic syndrome (Ludwig et al., 2017; Panagiotidou et al., 2018; Wang et al., 2016).

It has been reported that up to 70% of patients with leptospirosis globally do not seek medical care due to asymptomatic presentation or mild symptoms that often mimics other diseases. Leptospirosis can mimic acute respiratory infections, acute surgical pathology, sepsis, or hemorrhagic fevers (Becirovic et al., 2020; Izurieta et al., 2008; Tubiana et al., 2013; Wickramasinghe et al., 2019). In mild cases requiring treatment, the typical course of action is oral antibiotics such as doxycycline, azithromycin, or ampicillin. In severe cases, intravenous antibiotics such as penicillin or ceftriaxone are initiated (WHO, 2003) and the fatality rate is high, with reported ranges of 5–15%. On average, 60,000 patients die from leptospirosis worldwide every year (CDC, 2018; Costa et al., 2015).

Historically, numerous tests have been used for diagnosis of leptospirosis. A serological test, the Microscopic Agglutination Test (MAT), is the gold standard for leptospirosis diagnosis with an accuracy of ∼75% to 80% (Goris and Hartskeerl, 2014). The sensitivity of the assay increases from 41% if used during the first week of the illness to 96% if used during the fourth week of disease. Thus, this method often has only retrospective benefits for patients (Jayasundara et al., 2021; Musso and La Scola, 2013). In early stages of the disease, polymerase chain reaction (PCR) can be used to detect *Leptospira* in blood or urine samples. However, PCR is expensive and requires trained staff and additional equipment, and thus is not routinely used.

In addition, enzyme-linked immunosorbent assay (ELISA) can be used to differentiate Immunoglobulin M (IgM) and Immunoglobulin G (IgG) to *Leptospira*; however, this assay can also be cross-reactive to other spirochetal infections (*e.g.,* Lyme disease and syphilis) resulting in false positive results (CDC, 2018; Haake and Levett, 2015). Given the difficulty in a timely diagnosis of leptospirosis, the risk of lethal outcomes remains high. Thus, if leptospirosis is suspected, it is advised to initiate treatment even without a positive diagnosis. Furthermore, the medical community should be aware of the symptoms of leptospirosis even in nonendemic regions.

We describe a novel case of leptospirosis in a Ukrainian civilian. The disease is thought to be a direct consequence of the ongoing war in Ukraine due to unsuitable living conditions in bomb shelters and direct contact with dried remains of the excrement of rodents in the shelters. This forced change in lifestyle has created favorable environments for the emergence of numerous infectious diseases. In addition, this case presented without the characteristic prodromal period for leptospirosis adding to the complexity in diagnosis. This case should serve as a cautionary tale to medical professionals for heightened awareness of infectious disease outbreaks in nonendemic regions.

## Clinical Presentation

At the end of March of 2022, a 70-year-old man residing in Lviv became acutely ill with fever and jaundice, and was admitted to the First Medical Union Lviv Clinical Emergency Hospital (FMULCEH) on the suspicion of mechanical jaundice. Upon admission, the patient was alert, and his general status was moderate with an elevated temperature of 38.6°C. A physical examination revealed that his skin and mucosae appeared yellowish and his tongue was plastered with dirty fur. The posterior pharyngeal wall was yellowish and the tonsils were clear of any spots. Vesicular breathing was heard across the lung surface and his respiratory rate was 21 breaths per minute. The heartbeat was clear and regular with an average of 102 beats per minute. Upon palpitation, the abdomen was soft. Daily urine was sufficient and properly colored (straw-colored), and stool was formed and routine (one bowel movement per day). No meningeal signs or focal neurological symptoms were observed, and viral hepatitis and SARS-CoV-2 were ruled out.

**FIG. 1.**
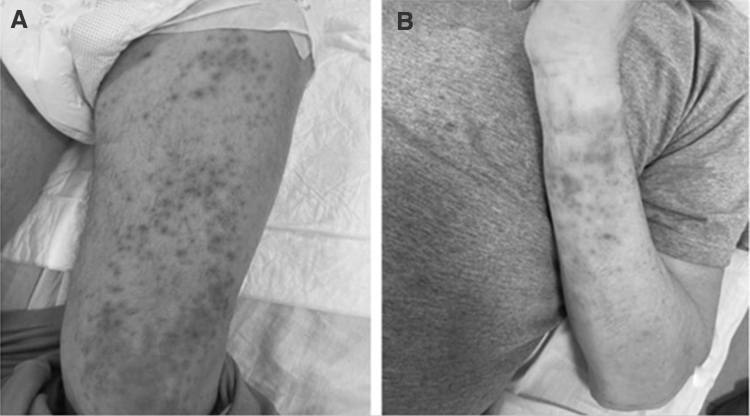
Appearance of a rash in a patient with leptospirosis. The rash developed on day 6 of the disease and was present on the thigh **(A)** and the forearm **(B)**.

After admittance into the hospital, additional tests were conducted, including abdominal CT and bloodwork (day 3 of the disease). The abdominal CT scan showed no signs of obstruction and thus the diagnosis of mechanical jaundice was eliminated. Blood and urine tests revealed renal insufficiency, which was evidenced by decreasing volume of diuresis and increasing levels of serum creatinine and urea (Table 1). The patient also developed thrombohemorrhagic syndrome as evidenced by thrombocytopenia and a large draining hemorrhagic rash on the extremities on day 6 (Fig. 1).

**Table 1.** Blood and Urine Test Results

Data	Patient values	Normal ranges
CBC		
Hb (g/dL)	**12.8**	13.2–16.6
Red blood cells ( × 10^12^/L)	4.34	3.8–5.8
Ht	0.373	0.35–0.5
White blood cells ( × 10^9^/L)	***32.9***	3.5–9.5
Granulocytes (%)	***93.4***	40–75
Lymphocytes (%)	***4.5***	20–40
Monocytes (%)	**2.1**	1–10
Platelets cells ( × 10^9^/L)	***79***	125–350
ESR (mm/h)	***42***	10–15
Urine test		
Color	***Very yellow***	Mild yellow
Red blood cells (in a field of view)	Not changed, single	Absent
White blood cells (in a field of view)	***23***	6–8
Protein	***Traces***	Absent
Epithelium (in a field of view)	Flat 1-2	Single
Alfa-amylase (U/L)	32.0	16–64
Biochemical tests		
Total bilirubin (μM)	***312.0***	2.0–21.0
Direct bilirubin (μM)	***90.04***	<6.8
ALT (U/L)	***95***	<45
AST (U/L)	***61.1***	<37
Alkaline phosphatase (U/L)	147.5	64.0–306.0
Urea (mM)	***24.1***	1.7–8.3
Creatinine (μM)	***179.0***	53.0–106.0
Coagulation test		
Prothrombin time (“)	**14.1**	10–14
Prothrombin index (%)	**79**	80–100
Fibrinogen (g/L)	***7.43***	2–4

Blood and urine tests were conducted on day 3 of disease. Values that are bolded are slightly out of normal ranges although not of concern. Values that are bolded italics are considered significantly out of normal range.

A detailed social history was conducted to assess potential infectious disease exposure to aid in diagnosis of this patient. Based on the epidemiological history, the patient lived alone and rarely left his apartment. Any living necessities were provided directly to him by his children. He had been living in his flat for many years but had not visited the basement before the start of the military conflict in February. Starting in February, the patient only left his house during air alarms and went to the bomb shelter located in the basement of his apartment complex.

During each of these alarms he spent an average of 1 h in the basement. In February and March in Lvivska Oblast were a total of 73 air alarms with an average duration of 70 min (Air-alarms.in.ua, 2022). According to the patient, the basement was dirty and cluttered and not intended to serve as living quarters. Per the patient, pets were not brought into the basement and there was no consumption of food or beverages during the alarms. No samples were procured from the basement for further testing.

Based on the clinical examination, blood tests, and social history, leptospirosis was suspected. Given the suspicion of leptospirosis, therapy was initiated before receiving confirmation of a leptospirosis diagnosis. Viral hepatitis B, C, and sepsis were considered in the differential diagnosis of leptospirosis. On day 4, the patient was administered intravenous meropenem due to the presence of hectic fever and hyperleukocytosis, and drip infusions (5% glucose, 0.9% NaCl) for the purpose of detoxication. In addition, the patient was prescribed furosemide, prednisolone, omeprazole, and etamzilat.

Owing to technical limitations in obtaining the MAT (the Ministry of Health of Ukraine suspended all high-threat live pathogen work after the start of the military conflict in Ukraine), the diagnosis of leptospirosis was confirmed on day 7 with an ELISA assay (SERION ELISA classic Leptospira IgM): increased titer of IgM antibodies (0.556 IgM) to *Leptospira interrogans,* reference negative values of IgM in this test system was <0.38.

The final diagnosis was icteric leptospirosis complicated by acute renal failure and pneumonia. After laboratory confirmation of the diagnosis of leptospirosis, the patient was transferred on day 8 to Lviv Oblast Clinical Hospital of Infectious Diseases (LOCHID). After admission to LOCHID, an x-ray revealed bilateral pneumonia. Therapy was intensified resulting in recovery from the disease. The patient was discharged on day 26 of the disease with normal complete blood count (CBC) and blood chemistry results.

## Discussion

In this study, we report a unique clinical case of leptospirosis in Ukraine. The patient had a severe case of leptospirosis with intensive jaundice, acute renal failure, and thrombohemorrhagic syndrome, and lung involvement. He was treated with antibiotics and fully recovered. This case is uniquely characterized by a lack of prodromal period, which is usually pronounced in leptospirosis. The new epidemiological circumstances in Ukraine, likely due to the military conflict at the time of symptom onset, forced residents into bomb shelters that are uninhabitable. The presence of dried remains of the excrement of rodents along with inhalation of dust particles likely contributed to the emergence of this clinical case. Taken collectively, this case warrants heightened awareness by the medical and scientific community of the emergence of infectious diseases resulting from new epidemiological conditions.

Military conflict has historically played an important role in the spread of infectious diseases through different routes of transmission (airborne, oral-fecal, sexual, *etc.*) as military personnel often remain in closed quarters for extended periods of time (Hughes et al., 2007; Korzeniewski et al., 2015; Riddle et al., 2015). Taken collectively, it is unsurprising that leptospirosis outbreaks are common occurrences among military personnel. Historical descriptions of leptospirosis in conditions of hostility date back to 1917, in which Weil's disease—a severe form of leptospirosis—was reported in the members of British army located in Flanders. Additional cases of leptospirosis occurred in military personnel during WWI and WWII (Brightman, 2018; Forbes et al., 2012; Stokes et al., 1917).

More recently, in 2014, 2016, and 2017 there were leptospirosis outbreaks among military personnel in Okinawa, Japan (81 confirmed cases), Hulu Perdik Forest, Malaysia (12 confirmed cases) and Mount Lam Lam, Guam (8 confirmed cases), respectively (Brinker and Blazes, 2017; Dierks et al., 2018; Neela et al., 2019). In the recent conflicts in Afghanistan and Iraq, there have been a limited number of leptospirosis cases reported due to difficulties in obtaining diagnostic testing for confirmation of the disease due to active conflict (Neela et al., 2019; Pappas et al., 2008). These cases of leptospirosis in military personnel have largely been linked to both training exercises and active conflict scenarios.

Training exercises commonly take place in tropical and subtropical locations, which are considered hyperendemic for leptospirosis resulting in an increased risk for outbreaks. These regions include but are not limited to Kuala Lumpur, Sri Lanka, Japan, Guam, and Malaysia (Agampodi et al., 2011; Schneider et al., 2012). Despite the well-described cases of infectious disease in military personnel around the world, limited work has been focused on transmission of diseases to civilians during military conflicts. In one of the systematic reviews of the literature, which analyzed 210 articles published from 1810 to 2020, only 17% of the works studied the morbidity of the civilian population (Chaufan et al., 2021).

Importantly, to our knowledge there have not been cases of leptospirosis among civilians during periods of conflict, and thus our case report is unique. One of the important aspects of the described clinical case is that the patient presented without a prodromal period. In typical cases of leptospirosis, patients present with a prodromal period that is often characterized by numerous symptoms, including intensive chills, myalgia, diarrhea, vomiting, cough, headache, and scleritis prior the onset of jaundice and renal involvement on days 4–5 of the disease (Levett, 2020).

In the case described in this study, none of these symptoms were present as jaundice and fever were the only presenting symptoms. The lack of a pronounced initial period of the disease could be explained by high levels of stress due to the conditions of war, which may have suppressed awareness of other symptoms. This is important as the limited number of any leptospirosis symptoms in the prodromal period led doctors to suspect other illnesses, which resulted in delayed therapeutic interventions.

Owing to disruptions caused by the war, the Food and Agriculture Organization of the United Nations (FAO) issued a statement on the increased risk of infectious diseases, including but not limited to leptospirosis, tularemia, West Nile Fever, and Crimean-Congo Hemorrhagic Fever (CCHF). The FAO has made recommendations for initiatives within Ukraine that would inform public, military, and professional groups on the risks of cross-border diseases (FAO, 2022). Suggestions to mitigate outbreaks of zoonotic diseases include the establishment of multidisciplinary groups of experts to evaluate risk, monitor outbreaks, enhance disease reporting and detection, create early outbreak warning systems, and continue research efforts for vaccine development.

In addition, the World Health Organization (WHO) analyzed the situation in Ukraine and issued an analytical document with recommendations for the prevention of infectious diseases, including leptospirosis (WHO, 2022b). Thus, given the depth of the humanitarian catastrophe that has arisen in Ukraine because of the ongoing military conflict, there will likely be a rise in the incidence of leptospirosis and other infectious diseases.

## Conclusions

This article describes a novel case of leptospirosis in a Ukrainian civilian. This case arose during active military conflict. Owing to the lack of full-fledged bomb shelters in Ukraine, civilians have sought shelter in basements of multiapartment buildings. An epidemiological investigation of this case suggests that the most likely location of infection was in the basement through household contact with rodents (gray rats). Unfortunately, due to the ongoing conflict in Ukraine no samples were collected and tested from the basement to confirm these hypotheses. Future preventable measures could include extensive pest control in bomb shelters to reduce the presence of rodents as these are vectors for numerous infectious diseases, including leptospirosis.

Importantly, this case did not exhibit a typical course for leptospirosis, as the prodromal period lacked obvious symptoms of leptospirosis, which initially ruled out suspicion of leptospirosis until subsequent testing confirmed the diagnosis. Fortunately, because of timely therapeutic strategies the patient made a full recovery. Although described as a neglected zoonotic disease, the increased incidence of leptospirosis is likely because of the situation that developed in Ukraine warranting special attention and warnings from doctors regarding the emergence of infectious diseases under these new living conditions.
